# Morphological structure of salivary glands, alimentary canal, and Malpighian tubules in adult *Eurydema spectabilis* Horváth, 1882 (Heteroptera, Pentatomidae)

**DOI:** 10.1002/jemt.24684

**Published:** 2024-10-02

**Authors:** Hicret Arslan, Selami Candan

**Affiliations:** ^1^ Science Faculty, Department of Biology Gazi University Ankara Turkey

**Keywords:** alimentary canal, electron microscopy, light microscopy, Malpighian tubules

## Abstract

*Eurydema spectabilis* (Heteroptera: Pentatomidae) has a piercing‐sucking mouth type and feeds on plant sap. In this study, the morphological structure of the salivary glands, alimentary canal, and Malpighian tubules of *E*. *spectabilis* was examined using light and scanning electron microscopy. Salivary glands consist of the principal and accessory salivary glands. In *E*. *spectabilis*, digestion begins in the mouth and ends in the anus. Alimentary canal is divided into three parts: foregut, midgut, and hindgut. The foregut consists of pharynx, esophagus, and proventriculus. The esophagus connects to the proventriculus and resembles a narrow tube. The wall of the proventriculus has a recessed structure and is surrounded by a single cylindrical layer of epithelium and muscle. The midgut is divided into three regions: the first, second, and third ventricles (V1‐V3). V1 and V2 consist of single‐layered cylindrical epithelium. V3 contains a single layer of cuboidal epithelium. Gastric caeca were found in the midgut. The hindgut consisted of a pylorus followed by a well‐developed rectum. The wall of the rectum consists of a single‐layer cuboidal epithelium and muscle. Its lumen contains numerous bacteria and uric acid crystals. The pylorus consists of a single‐layered cylindrical epithelium. It is also the origin of Malpighian tubules. Malpighian tubules consist of two regions: proximal and distal. The morphological structure of the salivary glands, alimentary canal, and Malpighian tubules of *E*. *spectabilis*, which has not been studied before, was examined and discussed in comparison with other orders. It is also aimed to contribute to future studies.

**Research Highlights:**

- In *E*. *spectabilis*, the salivary glands are divided into principal and accessory salivary glands.
- Microvilli and numerous secretory granules were found in Malpighian tubules.
- Numerous uric acid crystals and bacteria were found in the rectum lumen.

## Graphical abstract

In *E. spectabilis*, the salivary glands are divided into principal and accessory glands. Microvilli and numerous secretory granules were found in Malpighian tubules. Numerous uric acid crystals and bacteria were found in the rectum lumen.

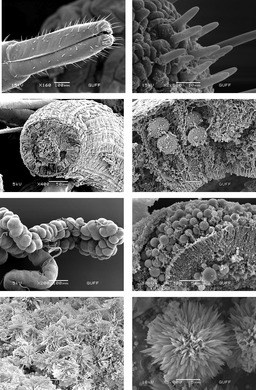

## 1 INTRODUCTION

Pentatomidae, one of the most populous families of the order Heteroptera, is very rich in terms of the number of species. Most of these species are of great importance because they are phytophagous and feed on a wide variety of wild plants (Bolu et al., 2006). Heteropterans that feed on plant sap use their piercing‐sucking mouthparts to pierce the plant and suck the plant's vascular fluid and sap. Some Heteroptera are also predators, feeding mainly on insects (Schaefer & Panizza, 2000).

The genus *Eurydema* Laporte, 1833 is classified in the Strachiini tribe of the Pentatominae subfamily (Rider, 2006). The species of this genus are generally colored white/yellow and black or red and blue and black. This aposematic coloration functions as a warning signal of toxicity even when the species possesses chemical or physical defenses to deter predators. Species of this genus are usually trophically bound to the plants of the family Brassi‐caceae and some of them, are known to cause damage to cultivated crops (Bohinc & Trdan, 2012).

Hemipteroidea have a piercing‐sucking mouth structure called “rostrum” or proboscis specialized for feeding (Demirsoy, 1997). It is natural that the rostrum structure differs in insects that feed on plant sap or blood (Dia et al., 2013). The structures on the rostrum, called sensilla, are sensitive structures that can interact with and detect food. These structures can be of different types and shapes. Thanks to the sensilla, insects can recognize the target area and feed easily (Anderson et al., 2006; Brożek & Chłond, 2010; Cobben, 1978; Gaffal, 1981; Parveen et al., 2015; Peregrine, 1972).

In insects belonging to the order Heteroptera, the alimentary canal starts with the mouth and ends with the anus, and digestion is divided into three parts. The foregut, which stores, filters, and partially digests nutrients; the midgut, where digestion and absorption take place; and finally the hindgut, which is responsible for the absorption of water and homeostasis. While the foregut and hindgut are covered with cuticle, the midgut lacks this structure (Candan et al., 2021; Dantas et al., 2021; Özyurt Koçakoğlu, 2021; Özyurt Koçakoğlu & Candan, 2021).

In insects, the foregut usually consists of the oral cavity, pharynx, esophagus, intestine, and proventriculus or gizzard (grinds food). Again, insects with the piercing‐sucking mouth type such as Heteroptera‐Hemiptera do not have a crop because they feed on plant sap (Özyurt Koçakoğlu, 2021). The middle intestine is usually the longest part of the intestine. It is also called the ventricle or stomach. Most of the digestion takes place here. The epithelial cells of the midgut produce digestive enzymes and the resulting nutrients and secretions are reabsorbed (Dadd, 1970; Gullan & Cranston, 2005; Sarwade & Bhawane, 2013; Wigglesworth, 1972). The heteropteran midgut is a long tube with morphological and functional differentiation; the midgut is the main site where digestion and absorption take place and where numerous digestive enzymes are released (Billingsley & Lehane, 1997; Chapman, 2013; Fialho et al., 2009).

Most insect groups have a sac at the anterior end of the midgut, called the gastric caeca. The gastric caeca is the main site where digestive enzymes are formed and digestive products are absorbed (Chapman, 1998). The hindgut is ectodermal in origin and lined with chitin (Maddrell & Gardiner, 1980; Özyurt Koçakoğlu et al., 2020; Sarwade & Bhawane, 2013; Sinha, 1958).

The hindgut is the last part of the alimentary canal in insects and is generally examined in three parts. They are ileum, colon, and rectum. Again, in Heteroptera, the hindgut consists of two parts, pylorus, and rectum (Gullan & Cranston, 2005; Özyurt Koçakoğlu, 2021).

In most insects, the rectum, rectal pads, and/or Malpighian tubules are structures responsible for specialized excretion and osmoregulation (Candan et al., 2021; Gullan & Cranston, 2005; Özyurt Koçakoğlu & Candan, 2022).

Malpighian tubules are long, thin tubular extensions that are closed at one end and consist of a single layer of cells. They are generally found free in the hemosol and filter solutes. It is responsible for removing nitrogenous wastes (ammonium ions) from hemolymph. Poisonous ammonium (NH₄) is rapidly converted into urea, then into uric acid, through a series of chemical reactions occurring within the Malpighian tubules, and finally discharged into the rectum (Chapman, 1998; Gullan & Cranston, 2012).

In the lumen of the hindgut, it reabsorbs water, salt, and necessary structures during the passage of waste materials coming from the Malpighian tubules and urine toward the anus (Chapman, 1998; Demirsoy, 2003; Gullan & Cranston, 2012). It is especially useful for insects living in the terrestrial region, as it reduces water loss in their bodies (Demirsoy, 2003).

Salivary glands, alimentary canal, and Malpighian tubules of *Eurydema spectabilis* (Pentatomidae) has not been investigated before. In this study, the histological and morphological structure of salivary glands, alimentary canal, and Malpighian tubules were examined and it was aimed to contribute this information to future studies.

## 2 MATERIALS AND METHODS

### 2.1 Insect and SM

Adult samples (male and female) of the present experimental beetle *E*. *spectabilis* (*n* = 20) were collected from an agricultural area at Kazan, Ankara, Turkey in June 2018. The samples were brought to the laboratory for light and scanning electron microscope (SEM) examination. First, the insects anesthetized with ethyl acetate fumes and dissected in 0.1 M sodium phosphate buffer, pH 7.2, were examined and photographed under Olympus SZX7 stereomicroscope (SM). Then, some of the specimens were fixed in 10% neutral formalin liquid for the light microscope (LM) and others in 2.5% glutaraldehyde (pH 7.2, sodium phosphate buffered) for SEM.

### 2.2 Light microscope

For histological observations, first, the alimentary canal of 10 samples was dissected and fixed in 10% neutral formalin liquid for 24 h. Then the specimens were washed in tap water and dehydrated from 50% to 100% in ethanol. Afterward, the samples were cleared up in 2 xylol series of 15 min each. The tissues were progressively changed from xylol to paraffin. Samples were definitively embedded in liquid paraffin at 65°C and then paraffin solidified at room temperature. The 5–6 μm thick sections were taken from these paraffin blocks by using a Microm HM 310 microtome. Sections taken from paraffin blocks were stained with Hematoxylin–Eosin (H&E) and Mallory 3 and photographed with Olympus BX51 LM.

### 2.3 Scanning electron microscope

For SEM examinations, 10 samples in 2.5% glutaraldehyde were first washed with sodium phosphate buffered (pH 7.2). They were then dehydrated in a graded ethanol series (from 50% to 100%). The samples were then transferred to hexamethyldisilazane (HMDS) and air‐dried. The dried samples were first photographed unbroken, then broken in certain places, attached to staples with double‐sided adhesive tape and coated with a thin layer of gold under vacuum in a Polaron SC502 coating device. The examinations were carried out with a JEOL JSM 6060 SEM at Gazi University and the photographs were taken at 5–15 kV.

## 3 RESULTS

### 3.1 Rostrum

**FIGURE 1:**
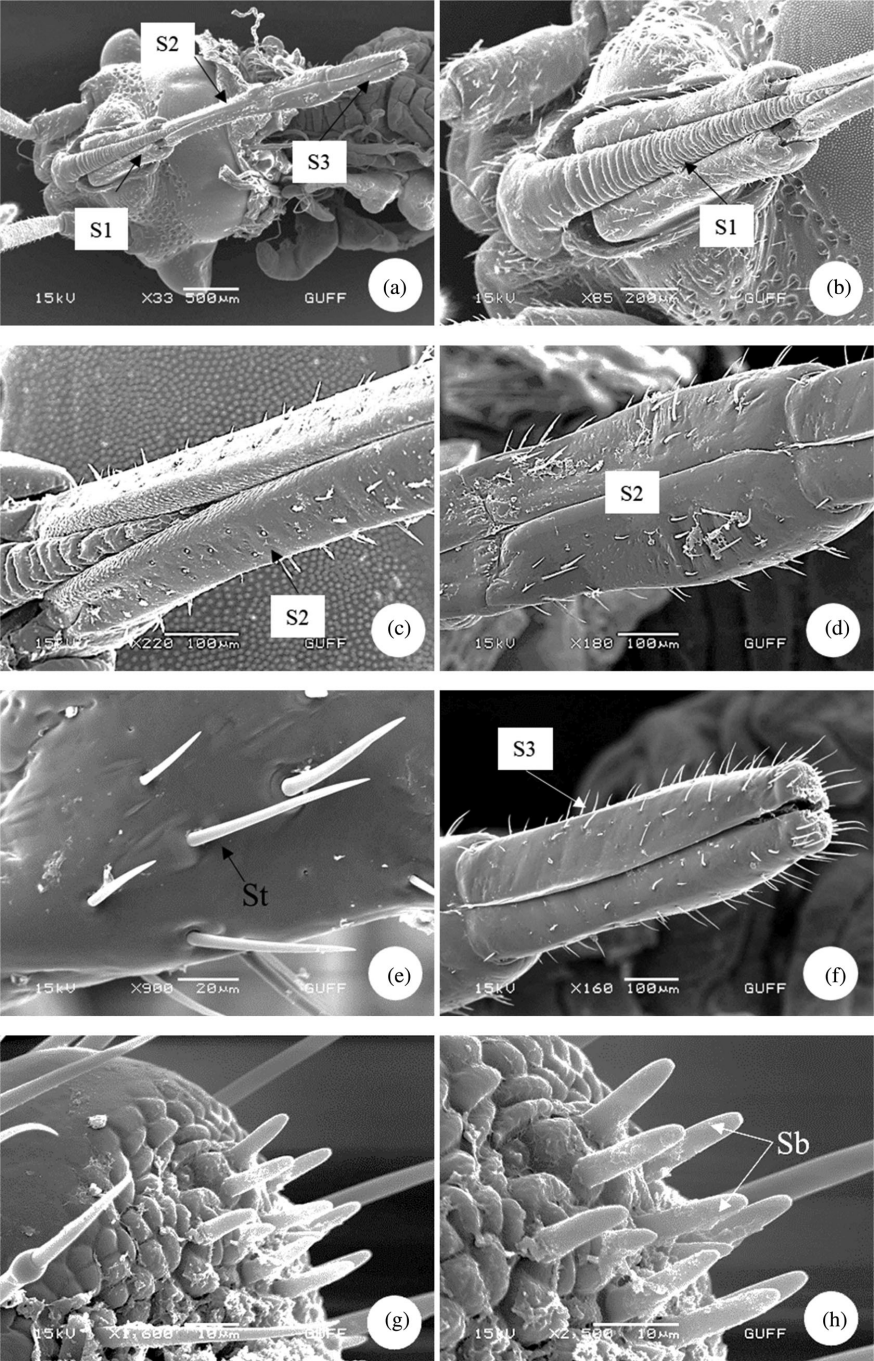
Rostrum (a) *Eurydema spectabilis* rostrum general view (scanning electron microscope). (b, c) General view of the rostrum segments and sensilla trichoidea (c–f) Rostum second and third segments and sensilla. (g, h) Sensilla basiconica at the tip. S1, S2, S3: segment; Sb, sensilla basiconica; St, sensilla trichodea.

**FIGURE 2:**
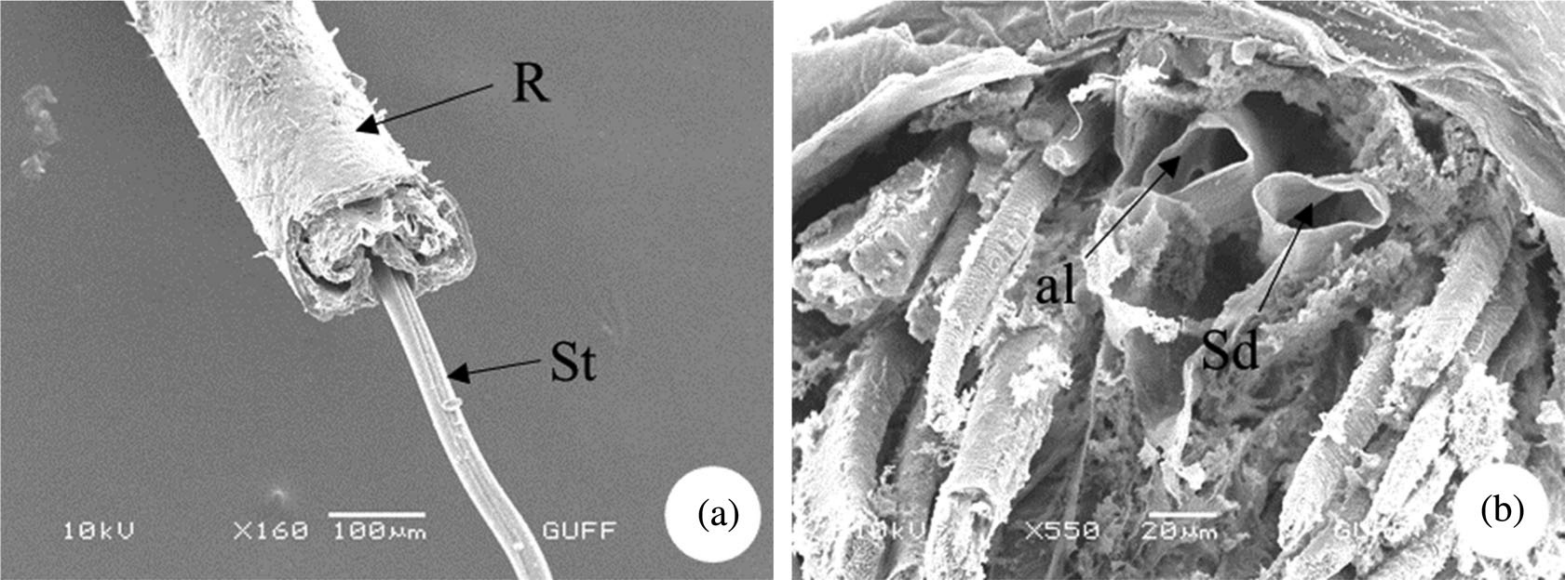
Ducts in the rostrum (a) Sucking tube. (b) Salivary duct and food duct (scanning electron microscope). al, alimentary canal; R, rostrum; Sd, salivary duct; St, sucking tube.

*E*. *spectabilis*, which feeds on plant sap, has a rostrum with a piercing‐sucking mouth structure for feeding. The rostrum consists of three parts from base to tip (Figure 1a–f). The segment from the base to the tip is ring‐shaped and allows the rostrum to move. The rostrum segments contain different types of sensilla (sensilla trichodea and basiconica), which are sensitive sensors that help locate food (Figure 1g,h). The mouth has a rostrum containing a long suction tube (Figure 2a). There is a long suction tube toward the end of the rostrum to draw the food in. SEM images show the presence of food channels (Figure 2b).

### 3.2 Salivary glands

**FIGURE 3:**
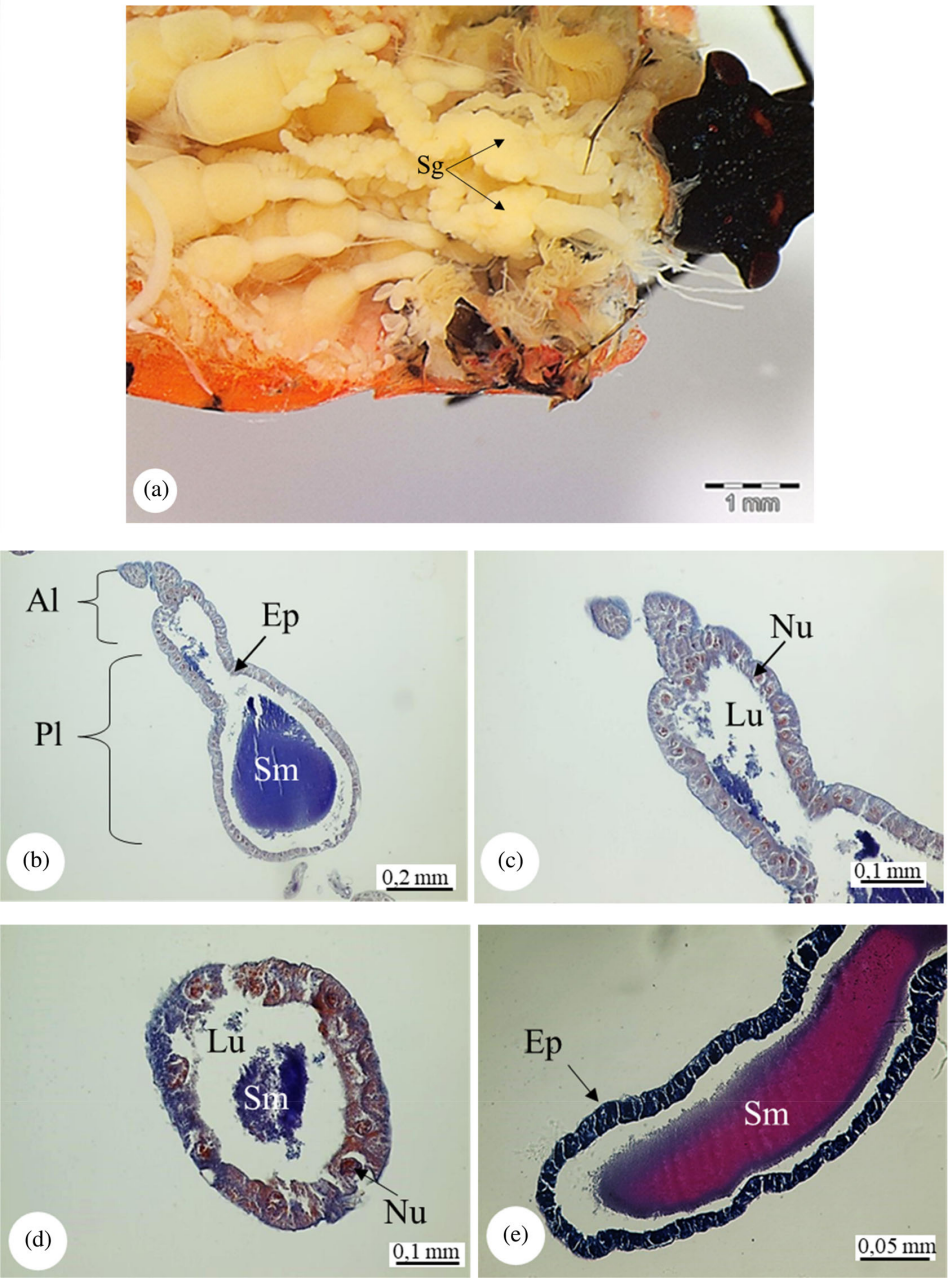
Salivary glands (a) Salivary gland general view (SM). (b) Salivary gland longitudinal section, anterior lobe and posterior lobe, (H&E), (LM). (c, d) Salivary gland cross section, lumen filled with secretion (H&E), (LM). (e) Salivary gland longitudinal section, (H&E), (LM). Al, anterior lobe; Ep, epithelium; Lu, lumen; Nu, nucleus; Pl, posterior lobe; Sg, salivary gland; Sm, secretory material.

*E*. *spectabilis* has a pair of salivary glands (Figure 3a). Histologically, the anterior and posterior lobes of the main salivary gland are similar (Figure 3b). The salivary gland wall is surrounded by a single row of cuboidal cells. Epithelial cell cytoplasm is basophilic. The cuticle covering of the lumen of the main salivary gland is absent. Secretory tissue is acidophilic and nongranular. There are large and round nuclei in the middle of each cell (Figure 3c–e).

**FIGURE 4:**
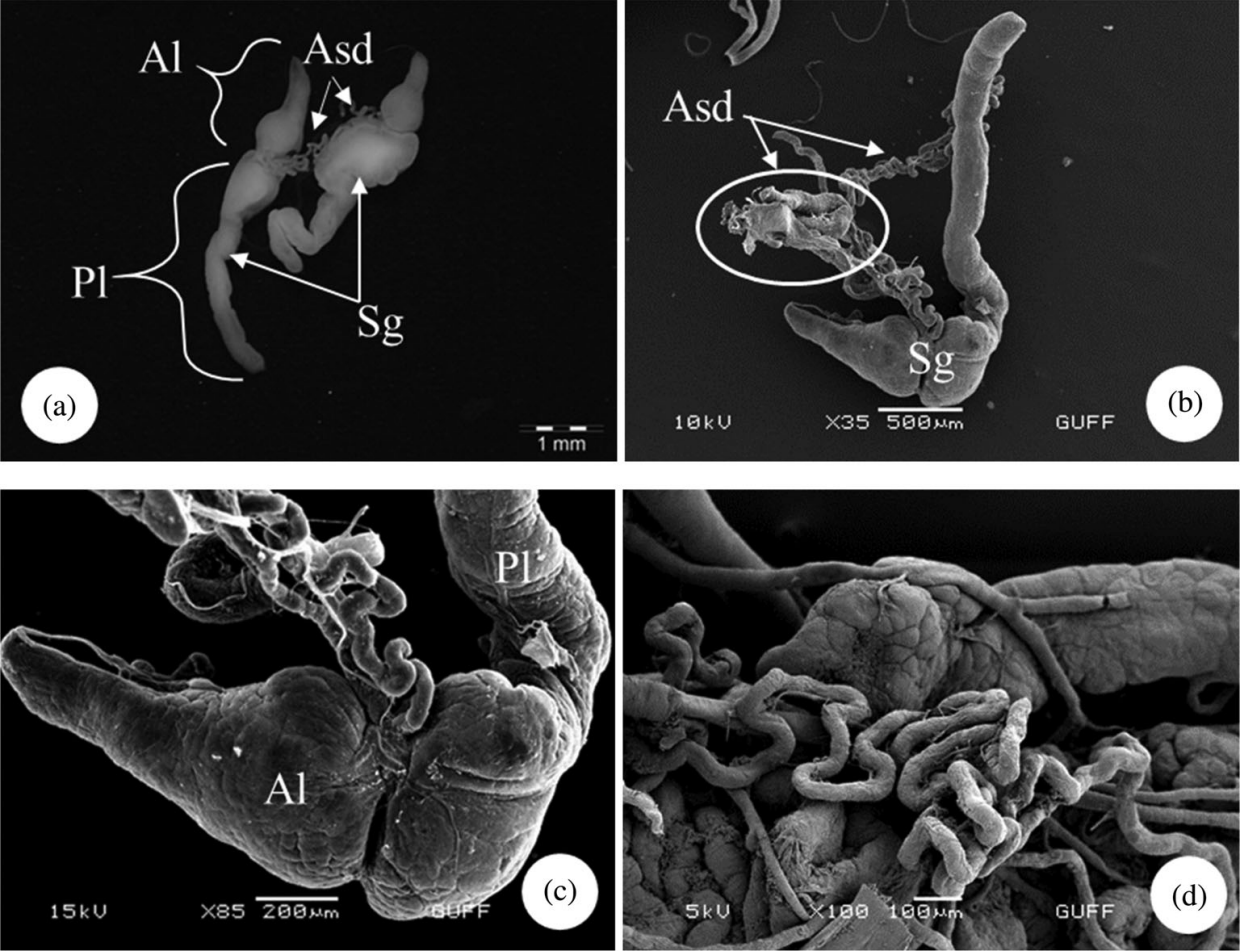
Salivary gland and accessory salivary ducts (a) General view of salivary gland and accessory salivary ducts, anterior lobe and posterior lobe (SM). (b–d). Salivary gland and accessory salivary gland duct view (scanning electron microscope). Al, anterior lobe; Pl, posterior lobe; Sg, salivary gland; Asd, accessory salivary duct.

**FIGURE 5:**
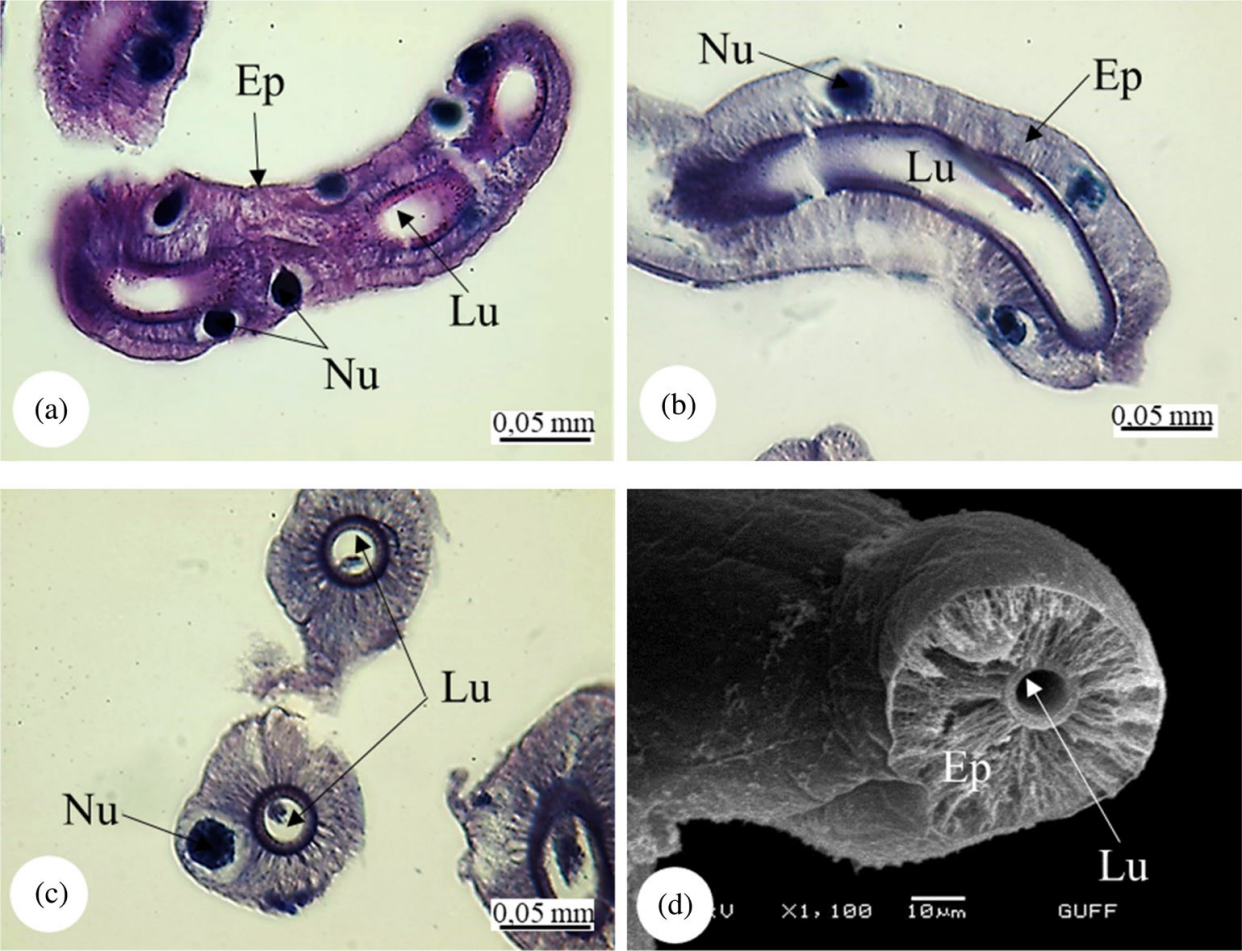
Accessory salivary gland duct (a, b) Accessory salivary gland duct longitudinal section. (H&E), (LM). (c) Accessory salivary gland duct cross section. (H&E), (LM). (d) Accessory salivary gland duct cross‐section (scanning electron microscope). Ep, epithelium; Lu, lumen; Nu, nucleus.

**FIGURE 6:**
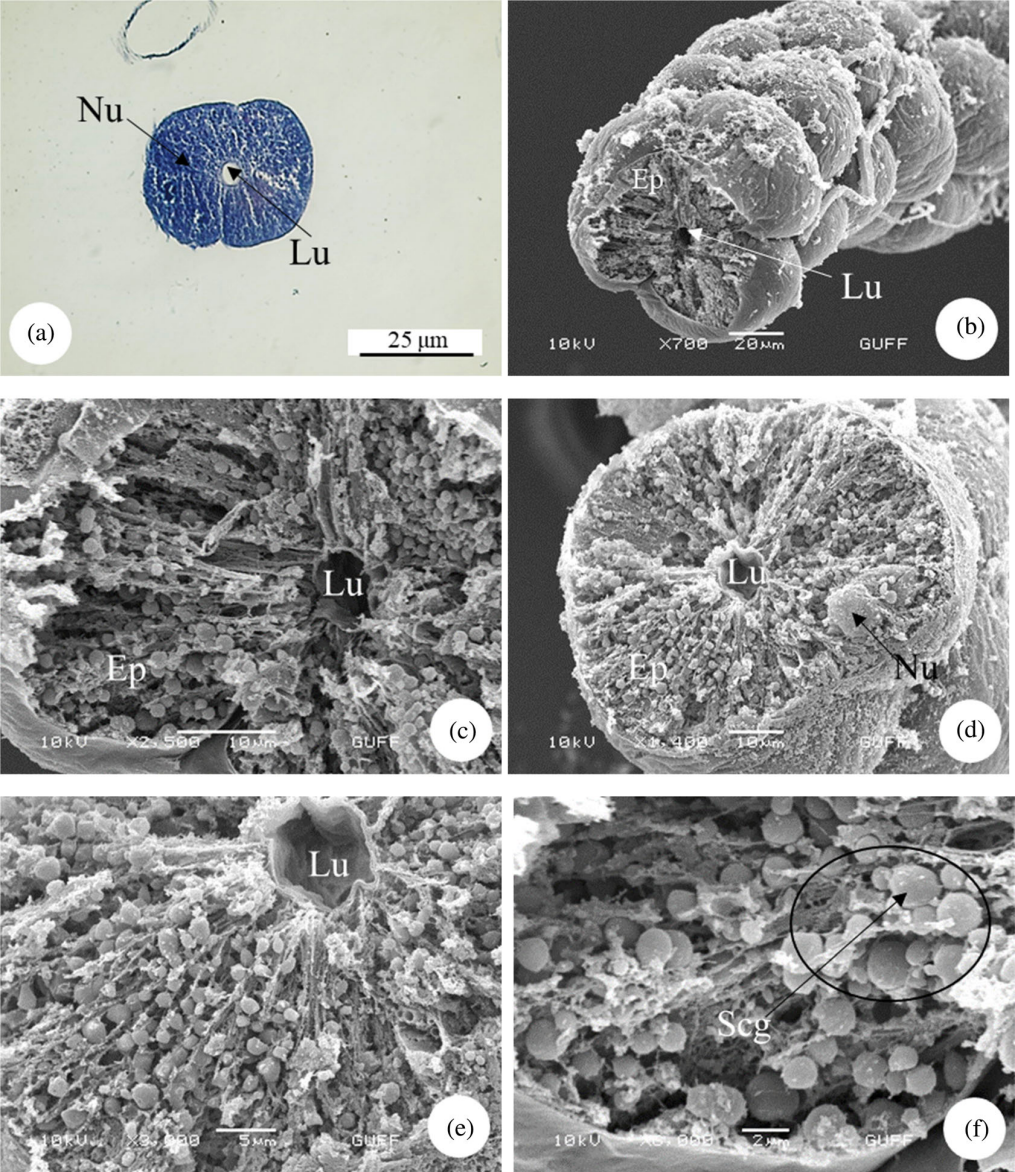
Accessory salivary gland (a) Accessory salivary gland cross section. (H&E), (LM). (b–d) Accessory salivary gland cross‐section (scanning electron microscope, SEM). (e, f). Abundant secretory granules (SEM) present in the accessory salivary gland. Ep, epithelium; Lu, lumen; Nu, nucleus; Scg, secretory granules.

*E*. *spectabilis* has an accessory salivary gland attached to each of the two salivary glands (Figure 4a,b). Scanning electron images show that the accessory salivary glands are long and wavy (Figure 4c,d). When LM and SEM images are examined, the lumen of the accessory salivary gland duct is quite narrow and its wall is surrounded by a single row of cuboidal cells and the wall is surrounded by a thick intima. There are large and basophilic nuclei in the middle of the cells (Figure 5a–d). The wall of the accessory salivary gland is surrounded by a monolayer cubic epithelium (Figure 6a). We also encountered the same image in SEM images (Figure 6b). The epithelial structure and lumen structure are quite clear (Figure 6c–e). Abundant secretory granules were found in the lumen (Figure 6f).

### 3.3 Alimentary Canal

**FIGURE 7:**
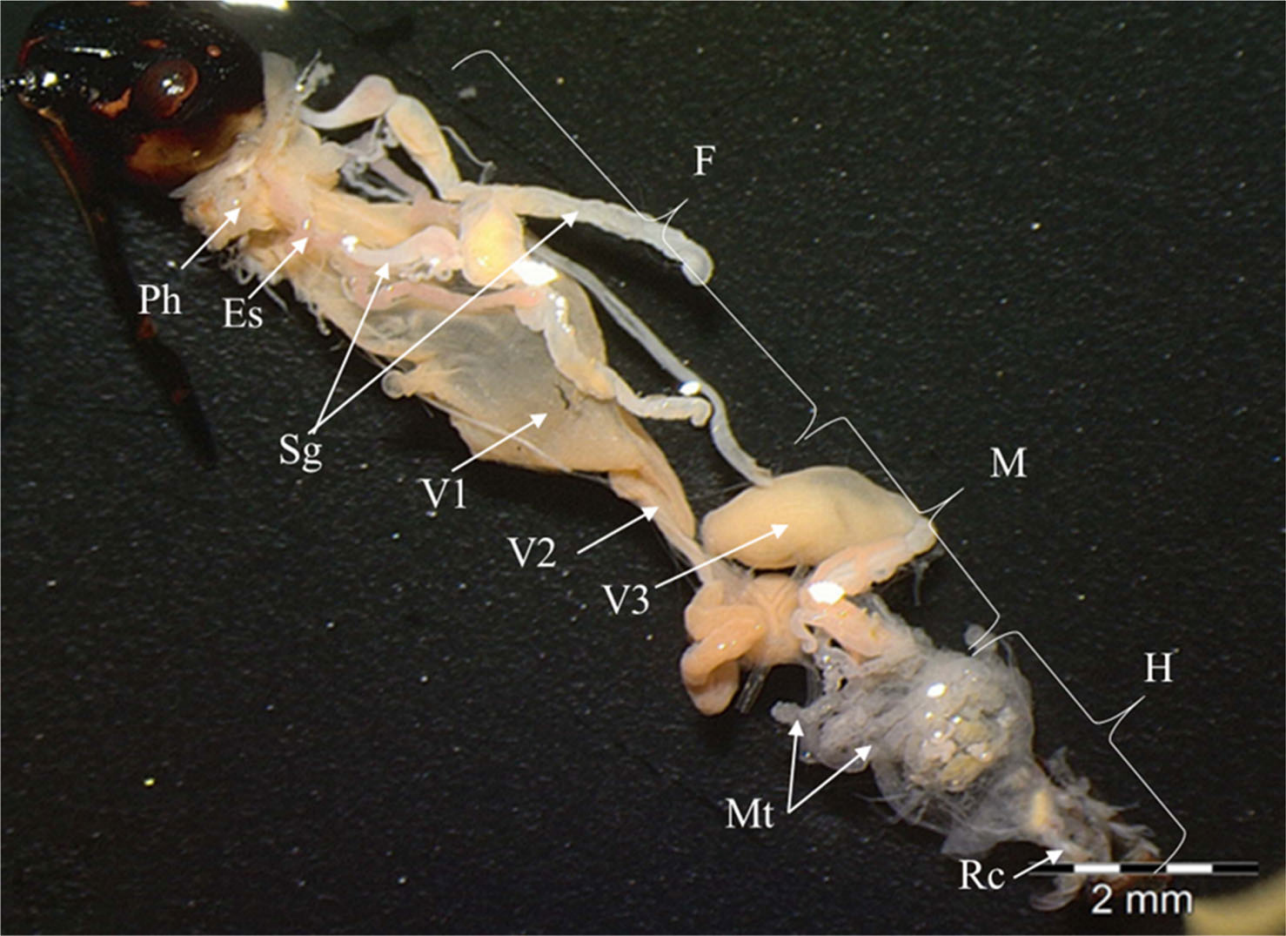
*Eurydema spectabilis* alimentary canal overview (SM). Es, esophagus; F, foregut; Gç, gastric caeca; H, hindgut; M, midgut; Mt, Malpighian tubule; Ph, pharynx; Rc, rectum; Sg, salivary glands; V1, Ventriculus 1; V2, Ventriculus 2; V3, Ventriculus 3.

In *E*. *spectabilis*, the alimentary canal begins with the mouth and ends with the anus. The alimentary canal of *E*. *spectabilis* is divided into three parts: Foregut, midgut, and hindgut. The foregut consists of the pharynx, esophagus, and proventriculus. The midgut is divided into three ventricles. The hindgut consists of the pylorus (ampulla) and rectum (Figure 7).

### 3.4 Foregut

**FIGURE 8:**
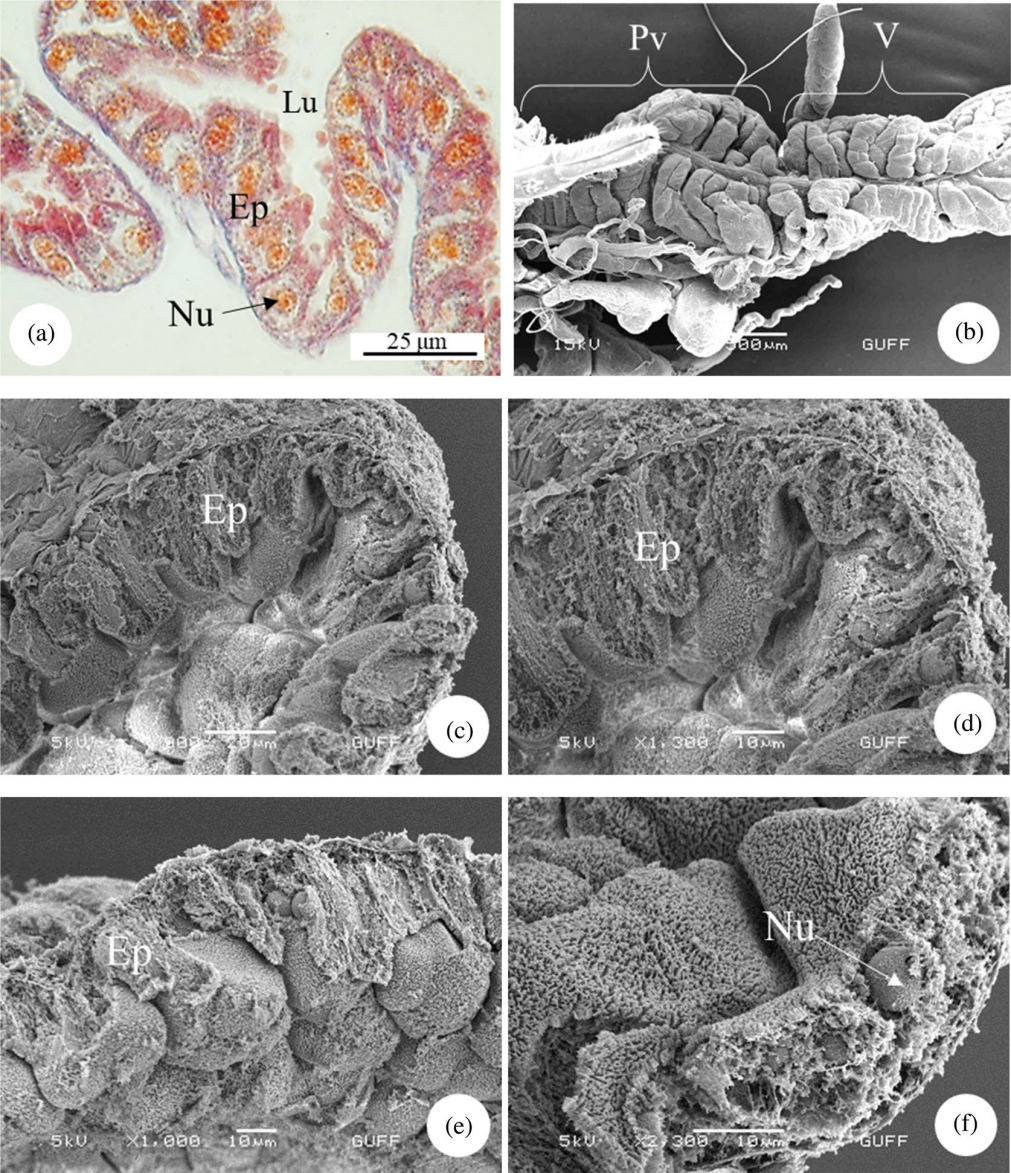
Proventriculus (a) proventriculus longitudinal section. (Mallory'3), (LM). (b) scanning electron microscope photograph of proventriculus and ventriculus (c–f). İnner surface structure of the proventriculus and nuclei embedded in the epithelium. Ep, epithelium; Lu, lumen; Nu, nucleus; Pv, proventriculus; V, ventriculus.

The foregut is the first part of the alimentary canal and consists of the pharynx, esophagus, and proventriculus. The proventriculus is located between the esophagus and the anterior midgut. When histological sections are examined, it is seen that the proventriculus wall consists of a single layer of cylindrical cells and is indented toward the lumen. Large and basophilic nuclei were found in the center of the epithelial cells (Figure 8a). Additionally, SEM images show that the proventriculus and ventriculus are clearly separated from each other (Figure 8b). When the SEM images are examined, it is seen that the junction points of the single‐layer cells of the rough structure are hollow and their middle parts rise apically, resulting in the emergence of a structurally curved structure (Figure 8c,d). The proventriculus wall has a recessed structure and is surrounded by a single‐layered cylindrical epithelium. Nuclei embedded in the epithelium were also found (Figure 8e,f). In general, the proventriculus and midgut have similar properties and are responsible for the digestion of food.

### 3.5 Midgut

**FIGURE 9:**
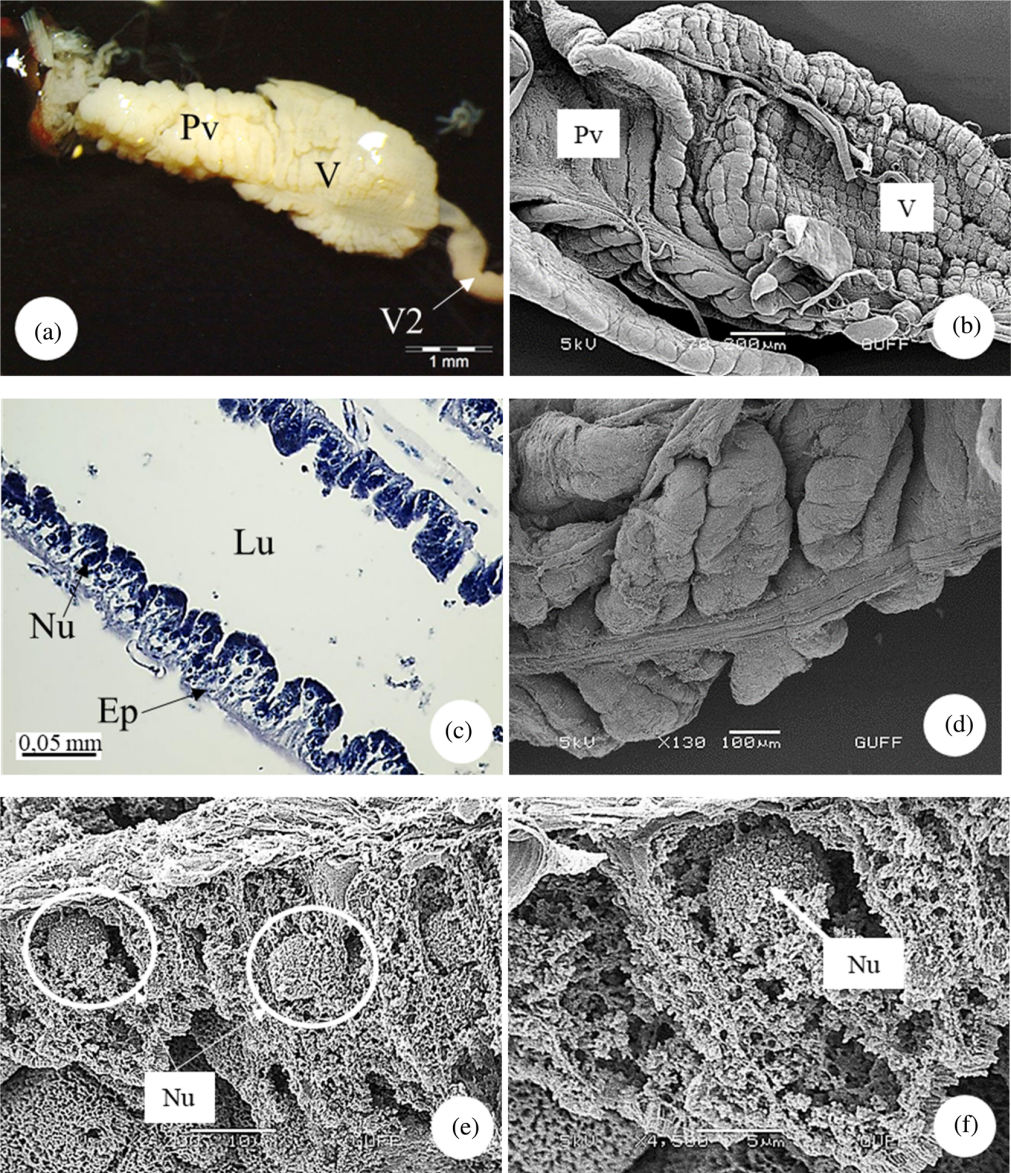
Proventriculus and midgut‐first ventricle (a, b) general view of proventriculus and first ventricle (SM‐scanning electron microscope, SEM). (c) Single‐layered cylindrical epithelial cell of the first ventricle structure. (H&E), (LM). (d–f) Ventricle cross‐section, single‐layered cylindrical epithelial sheet, and nuclei (SEM). Ep, epithelium; Lu, lumen; Nu, nucleus; Pv, proventriculus; V1, ventriculus 1; V2, ventriculus 2.

**FIGURE 10:**
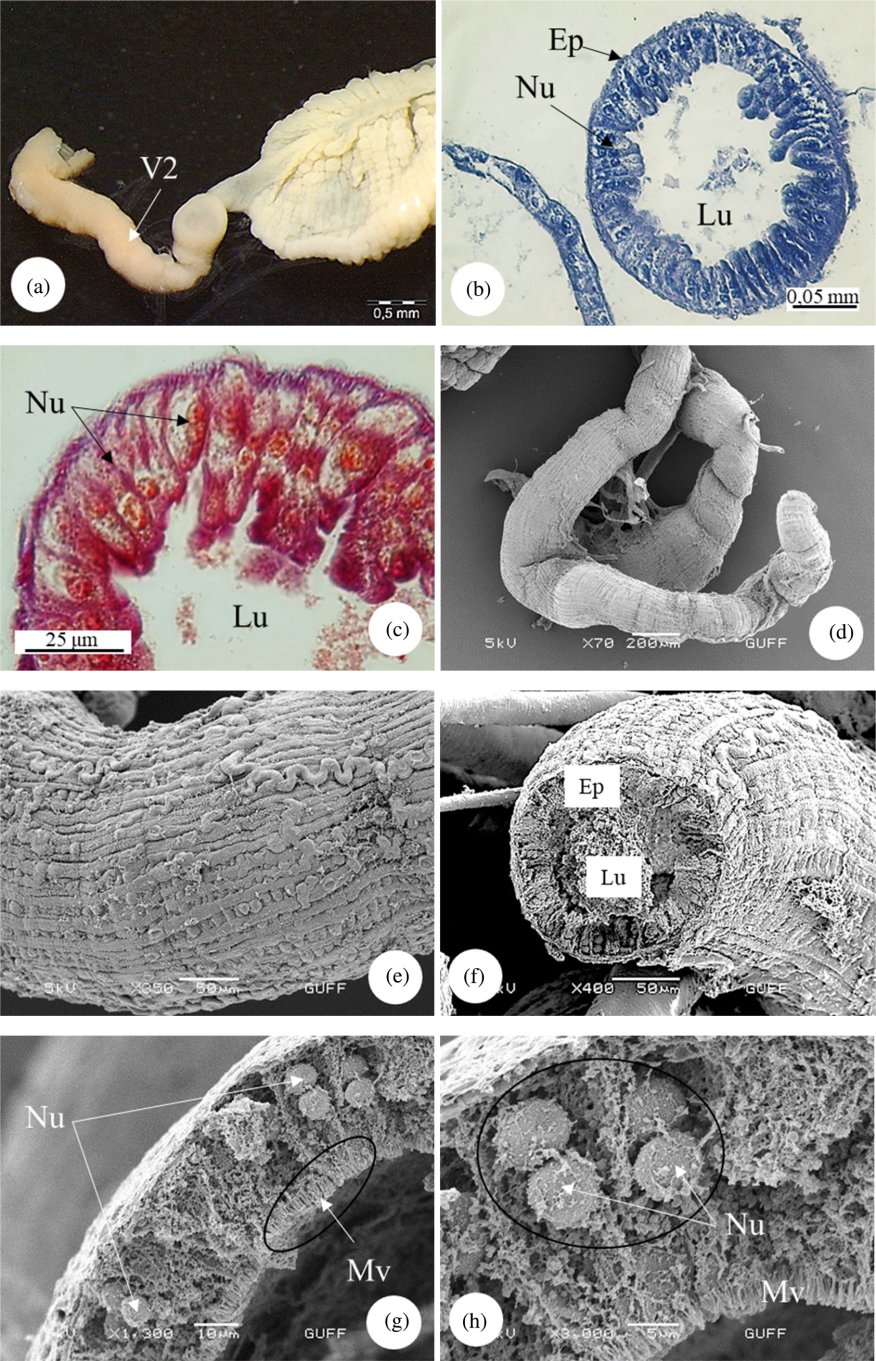
Midgut‐second ventricle (a) general view of the second ventricle of the stomach (SM). (b, c) Second ventricle monolayer cylindrical epithelial cells. (H&E)‐(Mallory'3), (LM). (d, e) Longitudinal muscles (scanning electron microscope, SEM) on the outer surface of the second ventricle. (f) Second ventricle cross section (SEM). (g, h) General view (SEM) of microvilli and nuclei in the second ventricle epithelium. Ep, epithelium; Lu, lumen; Mv, microvilli; Nu, nucleus; V2, ventriculus.

**FIGURE 11:**
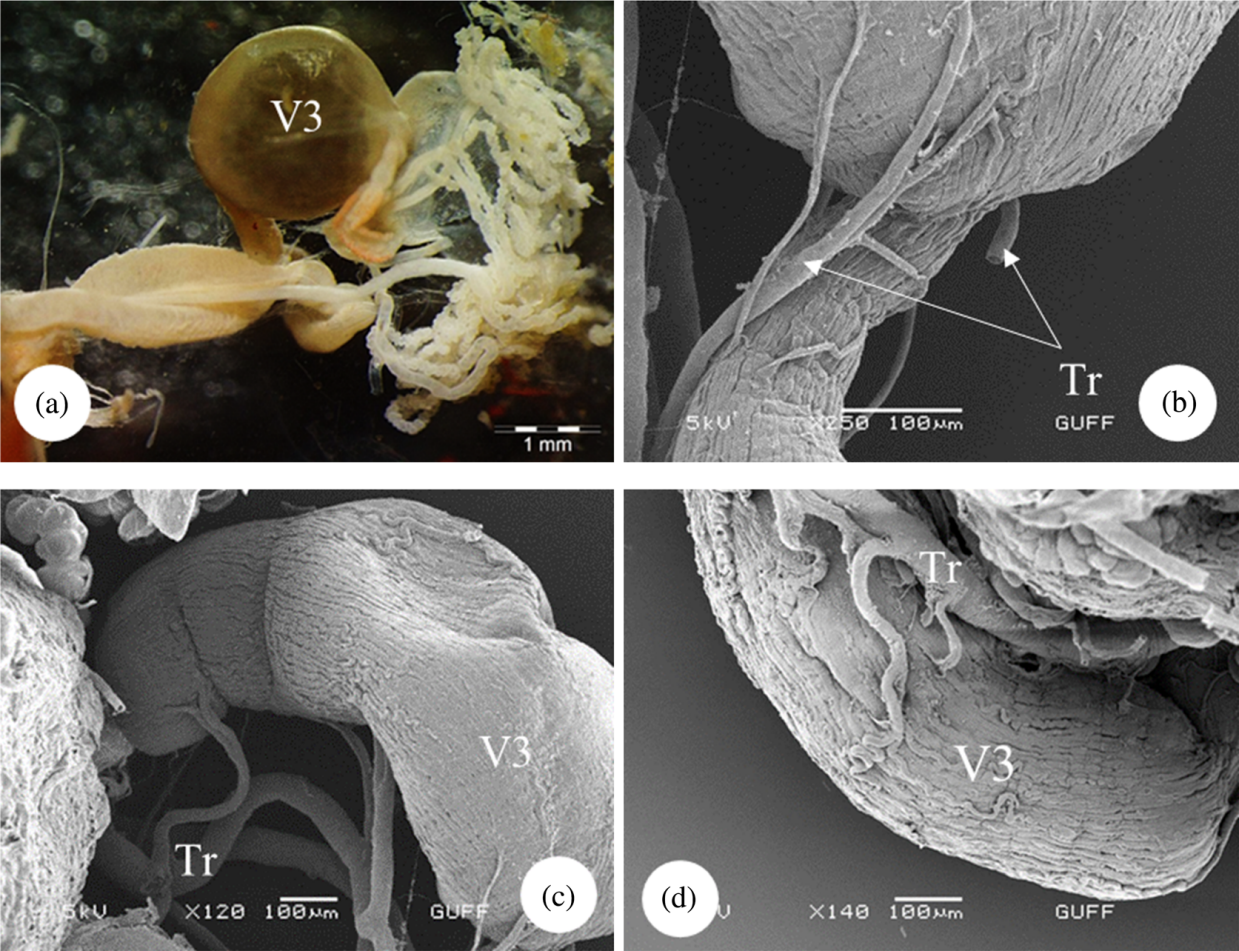
Midgut‐third ventricle (a) third ventricle general view (SM). (b–d) General view (scanning electron microscope) of the longitudinal muscles and tracheas on the outer surface of the third ventricle. Tr, trachea; V3, ventriculus 3.

The midgut is the longest part of the alimentary canal of *E*. *spectabilis* and is divided into three sections. The first ventricle is located in the continuation of the proventriculus (Figure 9a). SEM images show that the first ventricle is lobed and surrounded by tracheas (Figure 9b–d). In LM images, it was observed that the first ventricle had a wide lumen and consisted of a single layer of cylindrical cells. Nuclei are dispersed in epithelial cells (Figure 9c). In SEM examinations, it was observed that the first venticulus had a single‐layered cylindrical epithelial structure and nuclei embedded in the epithelium (Figure 9e,f). The second ventricle is in the form of a long tube that makes up approximately 2/3 of it and where digestion takes place (Figure 10a). In LM images, it appears that the structure of the second ventricle is circular and surrounded by a single layer of cylindrical cells. There is a narrow lumen and round and basophilic nuclei in the center of the cells (Figure 10b,c). In SEM images, it is seen that its outer surface is surrounded by longitudinal muscles (Figure 10d,e). A narrow lumen and a single cylindrical epithelial cell layer were observed in the sections (Figure 10f). Additionally, it was observed that there were nuclei embedded in the epithelium and microvilli on the side of the cells facing the lumen (Figure 10g,h). The third ventricle is a short, swollen balloon‐shaped structure (Figure 11a). Its function is to absorb excess water from food before it passes into the hindgut. In SEM images, it was observed that it was surrounded by longitudinal muscles and trachea on its outer surface (Figure 11b–d).

**FIGURE 12:**
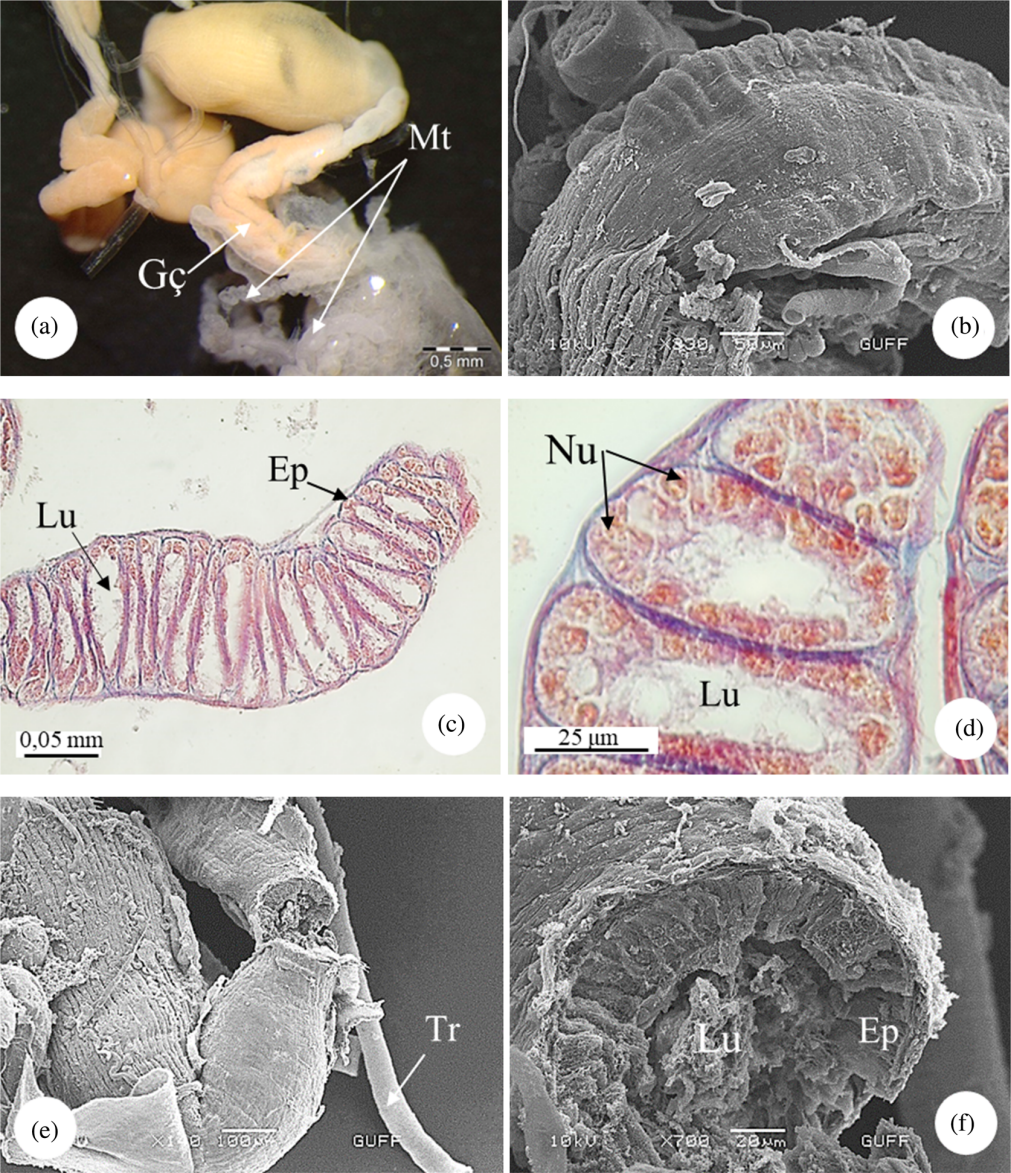
Gastric caeca (a, b) gastric caeca general view, (SM‐scanning electron microscope, SEM). (c, d) Gastric caeca monolayer cuboidal epithelial cells. (Mallory'3), (LM). (e, f) Gastric caeca trachea, cross‐sectional lumen and epithelial view (SEM). Ep, epithelium; Gç, gastric caeca; Lu, lumen; Mt, Malpighian tubule; Nu, nucleus.

The gastric caeca structure examined under the SM has a sac‐like structure. This structure, consisting of four channels, is connected to the midgut at one end and the hindgut at the other end (Figure 12a). In SEM images, the upper part of the structure is slightly wavy and the lower part is flat (Figure 12b). In LM images, the gastric cecum is shaped like a channel and consists of a thin epithelial cell wall. The lumen of the cecum structure, which consists of flat sacs, is quite narrow and consists of a single layer of cuboidal cells. Round nuclei embedded in the epithelium were found (Figure 12c,d). SEM images showed that the outer surface of the gastric caeca was smooth and surrounded by longitudinal muscles and trachea (Figure 12e). It was noticed in the SEM images that there was secretion in the lumen (Figure 12f).

### 3.6 Malpighian tubules and hindgut

**FIGURE 13:**
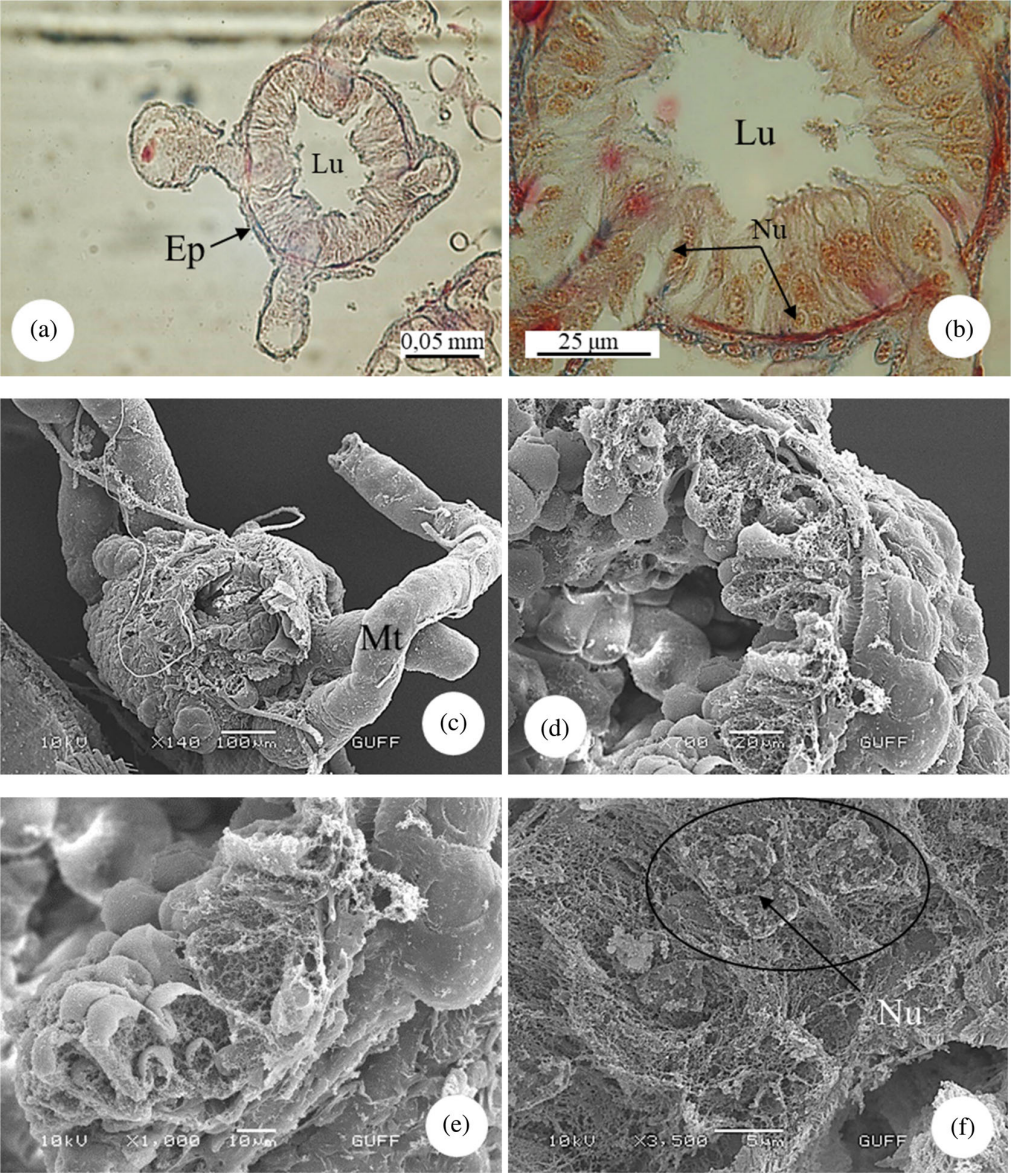
Pylorus (a, b) pylorus monolayer cylindrical epithelial cell. (Mallory'3), (LM). (c) Pylorus and Malpigi tubule general view, (scanning electron microscope, SEM). (d–f) Internal structure of the pylorus and general view of the nuclei (SEM). Ep, epithelium; Lu, lumen; Mt: Malpighian tubule; Nu, nucleus.

The pylorus is one of the parts of the hindgut where waste material and material from the Malpighian tubules are collected. In LM images, the pylorus structure is circular and the exit points of four Malpighian tubules are distinguished. The pylorus, which has a narrow lumen, consists of a single layer of cylindrical cells. Round nuclei were found in epithelial cells (Figure 13a,b). SEM images show the exit of Malpighian tubules from the pyloric region (Figure 13c). When the sample was broken and examined, it was seen that the internal structure was lobed (Figure 13d,e) and there were nuclei embedded in the epithelium (Figure 13f).

**FIGURE 14:**
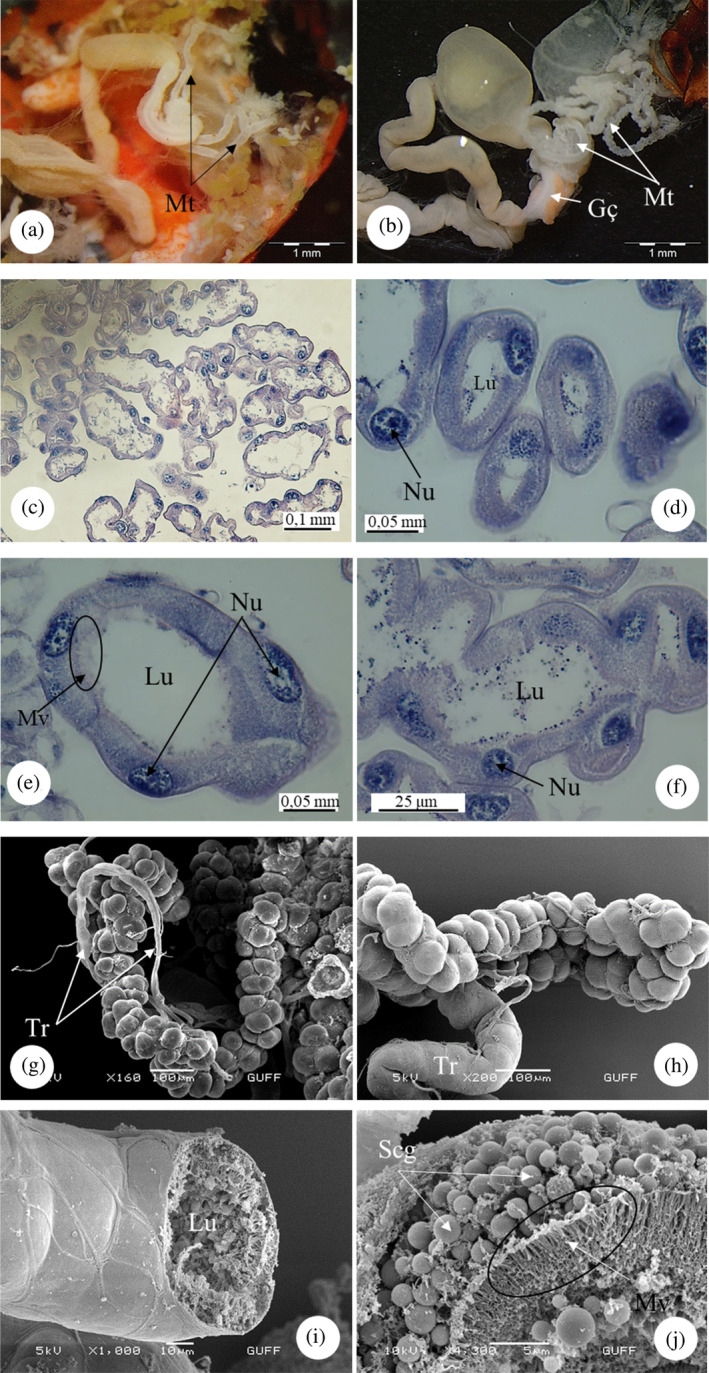
Malpighian tubules (a, b) Malpighian tubules general view, (SM). (c) Malpighian tubules longitudinal sections. (H&E), (LM). (d–f) Malpighian tubules cross sections, (LM). (g, h) Malpighian tubules general view, (scanning electron microscope, SEM). (i, j). Malpighian tubules cross‐section with secretory granules and microvilli, (SEM). Ep, epithelium; Gç, gastric caeca; Lu, lumen; Mt, Malpighian tubule; Mv, microvilli; Nu, nucleus; Scg, secretory granules; Tr, tracheae.

The pylorus, which forms the first part of the hindgut in the alimentary canal, is a triangle‐like structure with one end connected to the gastric caeca and two pairs of Malpighian tubules emerging from the other two ends (Figure 14a). It is distributed over the Malpighian tubules. The part connected to the pylorus is flatter, while the Malpighian tubules free in the body cavity are shaped like a string of beads (Figure 14b). In LM images, Malpighian tubules of *E*. *spectabilis* consist of a narrow lumen and a single layer of cuboidal cells. At the base of the cells are round or spherical shaped nuclei. Long microvilli structures with brush borders are evident in the parts facing the lumen (Figure 14c–f). SEM images revealed that the overall appearance was like a string of beads and wrapped around the trachea (Figure 14g,h). In SEM studies, when the Malpighian tubules was broken and examined, abundant secretory granules and microvilli were found (Figure 14i,j).

**FIGURE 15:**
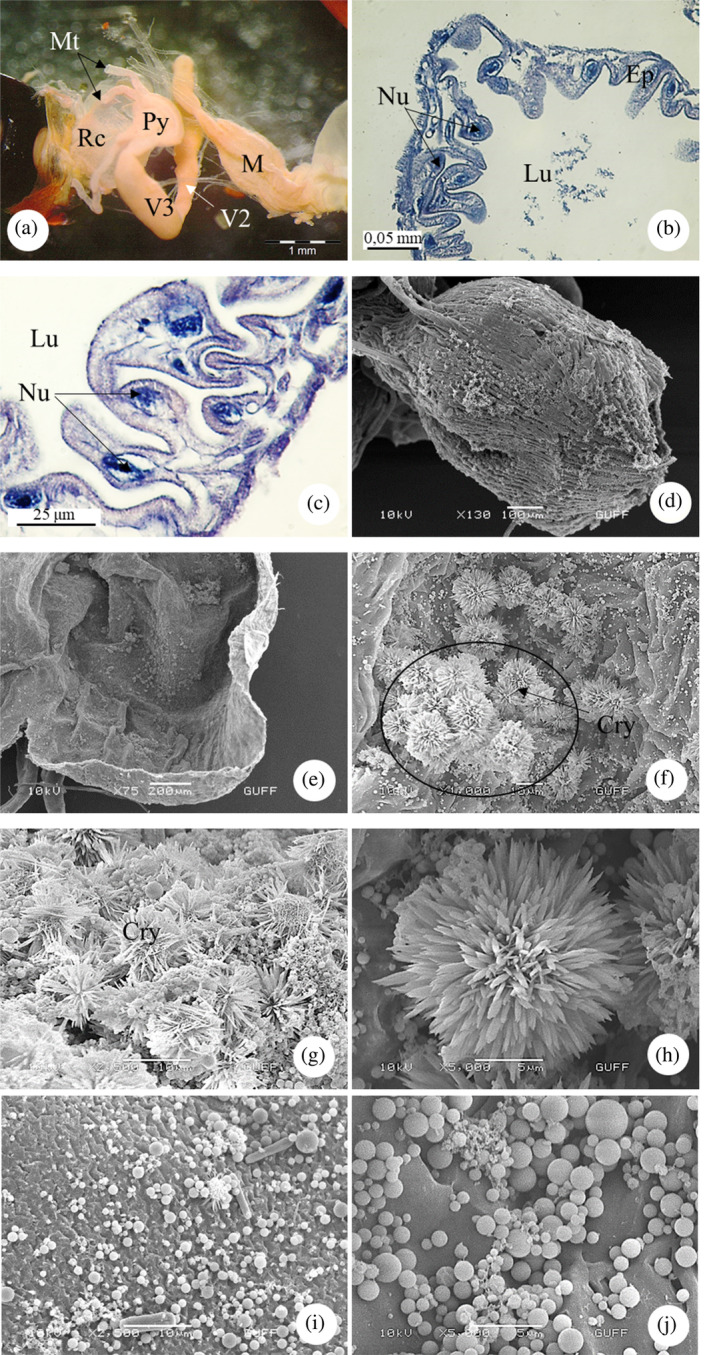
Rectum (a) rectum general view, (SM). (b, c) rectum cross‐sectional cuboidal epithelial cells. (H&E), (LM). (d, e) General view of inner and outer surface of the rectum (scanning electron microscope, SEM). (f–h). Uric acid crystals on the inner surface of the rectum, (SEM). (i, j) Bacteria on the inner surface of the rectum (SEM). Ep, epithelium; Gç, gastric caeca; Lu, lumen; M, midgut; Mt, Malpighian tubule; Mv, microvilli; Nu, nucleus; Py, pylorus; Rc, rectum; Scg, secretory granules; Tr, tracheae; V2–3, Ventriculus.

The rectum forms the last region of the hindgut (Figure 15a). Histologically, in LM images, the rectal wall is surrounded by a single layer of cuboidal cells and a muscle layer. Epithelial cells fold deeply towards the lumen and form a zigzag shape. Oval‐round nuclei were found in epithelial cells (Figure 15b,c). The surface of the rectum is surrounded by the trachea and longitudinal muscles (Figure 15d,e). When we examined the inner surface of the rectum, abundant uric acid crystals resembling flowers were detected (Figure 15f–h). Abundant bacteria were also found on the inner surface of the rectum (Figure 15i,j).

## 4 DISCUSSION

### 4.1 Rostrum

Rostrum segment numbers vary from species to species. The rostrum structure in *E*. *spectabilis* consists of three segments. However *Pyrrhocoris apterus* (Heteroptera: Pyrrhococoridae), *Graphosoma lineatum* (Heteroptera: Pentatomidae), *Coreus marginatus* (Heteroptera: Coreidae), *Gerris lacustris* (Heteroptera: Gerridae), and *Stephanitis nashi* (Heteroptera: Cimicomorpha) species are stated to consist of four segments (Gülsar, 2021; Wang et al., 2020a). Similarly, the rostrum segments of *Picromerus bidens* (Heteroptera: Asopinae), *Picromerus lewisi* (Heteroptera: Asopinae), and *Cazira bhoutanica* (Heteroptera: Asopinae) consist of four parts (Wang et al., 2020b).

In another study, *Chinavia hilaris* (Heteroptera: Pentatomidae), *Euschistus servus* (Heteroptera: Pentatomidae), it has been stated that the rostrum parts of *Oebalus pugnax* (Heteroptera: Pentatomidae) and *Piezodorus guildinii* (Heteroptera: Pentatomidae) species consist of four segments (Esquivel, 2019).

In *E*. *spectabilis*, the rostrum segments contain different types of sensilla (sensilla trichodea and basiconica). Rostrum structures were examined in *Eucryptorrhynchus scrobiculatus* and *Eucryptorrhynchus brandti* (Coleoptera: Curculionidae) and it was stated that there are different types of sensilla (Zhang et al., 2023).

### 4.2 Salivary glands

The salivary system of *E*. *spectabilis* has a pair of principal and accessory salivary glands and ducts, as in *Solubea pugnax* (Heteroptera: Pentatomidae), *Brontocoris tabidus* (Heteroptera: Pentatomidae), *Rhodnius prolixus* (Heteroptera: Reduviidae), *Carpocoris mediterraneus* (Heteroptera: Pentatomidae), and *Aelia rostrata* (Heteroptera: Pentatomidae) (Anhê et al., 2021; Azevedo et al., 2007; Carvalho et al., 2021; Genç & Candan, 2024; Hamner, 1936; Özyurt Koçakoğlu & Candan, 2022).

In *E*. *spectabilis*, principal salivary gland has two lobes as anterior and posterior lobes, as in *S*. *pugnax* (Heteroptera:Pentatomidae) (Hamner, 1936), *Scaptocoris castanea* (Heteroptera: Cydnidae) (Cossolin et al., 2019), *B*. *tabidus* (Heteroptera: Pentatomidae) (Carvalho et al., 2021), *C*. *mediterraneus* (Heteroptera: Pentatomidae) (Özyurt Koçakoğlu & Candan, 2022), and *A*. *rostrata* (Heteroptera: Pentatomidae) (Genç & Candan, 2024). However, in *Metacanthus elegans* (Heteroptera: Berytidae), *Chilacis typhae* (Heteroptera: Lygaeidae), and *Gastrodes ferrugineus* (Heteroptera: Lygaeidae), principal gland is trilobed with anterior, posterior, and lateral lobes (Baptist, 1941). Considering the differences between the orders, it is stated that the salivary glands of *Rhynshophorus ferrugineus* (Coleoptera: Dryophthoridae) are in the form of a long tube and consist of two main regions: reservoir and secretion (Norzainih et al., 2016). In some species, lobe numbers of principal salivary gland vary. *P*. *apterus* (Heteroptera: Pyrrhococoridae) have a quadrilobed principal gland as anterior, posterior, median, and lateral lobes (Baptist, 1941; Özyurt Koçakoğlu, 2021). The number of principal salivary gland lobes differs according to the species. However, such a situation was not observed in *E*. *spectabilis*.

In *E*. *spectabilis*, the principal salivary gland wall is surrounded by a single layer of cuboidal cells. Similar structure was found in *P*. *apterus* (Heteroptera: Pyrrhococoridae) (Özyurt Koçakoğlu, 2021), *C*. *mediterraneus* (Heteroptera: Pentatomidae) (Özyurt Koçakoğlu & Candan, 2022), and *A*. *rostrata* (Heteroptera: Pentatomidae) (Genç & Candan, 2024). However, in *Notonecta glauca* (Heteroptera: Notonectidae) each lobe of the pricipal gland has a single columnar layer (Baptist, 1941). They stated that the salivary glands of *Rhynshophorus ferrugineus* (Coleoptera: Dryophthoridae) consist of single‐layer epithelium and contain granules in the secretion zone (Norzainih et al., 2016).

The wall of the accessory salivary gland of *E*. *spectabilis*. It is surrounded by a monolayer of cuboidal epithelium and abundant secretory granules were found in the epithelium. Similarly, it was observed in *C*. *mediterraneus* (Heteroptera: Pentatomidae) (Özyurt Koçakoğlu & Candan, 2022). *P*. *apterus* (Heteroptera: Pyrrhocoridae) accessory salivary gland. It has a single layer of cuboidal epithelium. Secretory material was found in the lümen (Özyurt Koçakoğlu, 2021).

### 4.3 Alimentary Canal

The alimentary canal of *E*. *spectabilis*, which consists of the fore, mid, and hindgut is similar to the alimentary canal of other Heteroptera species (Hamner, 1936; Harris, 1938; Barber et al., 1980; Habibi et al., 2008; Çetin, 2014; Metin, 2014; Amutkan et al., 2015; Demirkol, 2016; Genç, 2017; Kara, 2020; Candan, Özyurt Koçakoğlu, Güllü & Çağlar, 2020; Özyurt Koçakoğlu, 2021).

### 4.4 Foregut and midgut

Proventriculus is a part of the foregut. In *E*. *spectabilis*, the proventriculus shows a structure with indented features in the wallpaper, resulting in the emergence of a single‐layered cylindrical epithelial structure. Nuclei embedded in the epithelium were found. Similar structures have been found in *Carpocoris pudicus* (Heteroptera: Pentatomidae), *Lygaeus equestris* (Heteroptera, Lygaeidae), *C*. *marginatus* (Heteroptera: Coreidae), *P*. *apterus*, and *A*. *rostrata* (Demirkol, 2016; Genç & Candan, 2024; Kara, 2020; Metin, 2014; Özyurt Koçakoğlu, 2021).

In some insect orders, teeth and spine‐like chitinous projections were found in the proventriculus. For example, eight chitinous tooth structures were observed in the proventriculus structure of *Tanymecus dilaticollis* (Coleoptera: Curculionidae) (Candan et al., 2020). Similarly, Orthoptera, Coleoptera and Hymenoptera that feed on solid foods such as *Melanogryllus desertus* (Orthoptera: Gryllidae), *Hypothenemus hampei* (Coleoptera: Curculionidae), and *Capnodis tenebrionis* (Coleoptera: Buprestidae) (Çakıcı, 2008; Özyurt Koçakoğlu et al., 2020; Rubio et al., 2008) have tooth‐like chitinous projections. As a result of these studies on the proventriculus, it is explained that the structure varies depending on solid and liquid nutrition. However, since *E*. *spectabilis* feeds on plant sap, no tooth‐like chitinous structures were found in the proventriculus.

The number of parts that make up the midgut varies between species. The midgut in *E*. *spectabilis* was examined in three parts. Similarly, *G*. *lineatum*, *Pyrops candelaria* (Heteroptera: Fulgoridae), *B*. *tabidus* (Heteroptera: Pentatomidae), *Cimex hemipterus* (Heteroptera: Cimicidae), and *Podisus nigrispinus* (Heteroptera: Pentatomidae) are divided into three ventricles (Amutkan et al., 2015; Azevedo et al., 2009; Cheung & Marshall, 1982; Guedes et al., 2003; Martínez et al., 2018). In *P*. *apterus* (Heteroptera: Pyrrhococoridae) and *C*. *mediterraneus* (Heteroptera: Pentatomidae) the midgut is divided into four ventricles. (Özyurt Koçakoğlu, 2021; Özyurt Koçakoğlu & Candan, 2022).

The shape of midgut epithelial cells varies between species. In *E*. *spectabilis*, midgut cells are arranged in a monolayer. The first and second ventricles consist of a single layer of cylindrical epithelium. Similarly, single‐layered cylindrical and cuboidal epithelial structures were found in *P*. *apterus* (Heteroptera: Pyrrhococoridae). While the first ventricle consists of cylindrical epithelium, the third and fourth ventricle consists of cuboidal epithelial layer (Özyurt Koçakoğlu, 2021). In *C*. *mediterraneus* (Heteroptera: Pentatomidae), the first and fourth ventricles consist of single‐layer epithelium, while the second and third ventricles consist of single‐layer cylindrical epithelium (Özyurt Koçakoğlu & Candan, 2022). In *Mylabris cernyi* (Coleoptera: Meloidae), the midgut epithelium is a layer of columnar cells covering a thin muscle layer, as most insects (Özyurt Koçakoğlu et al., 2022).

The gastric caeca, the last part of the midgut, is lined with a single‐layered epithelium in *E*. *spectabilis* and consists of flat sacs. Similar structures have been found in *Carpocoris pudicus* (Heteroptera: Pentatomidae), *Dolycoris baccarum* (Heteroptera: Pentatomidae), *Rhaphigaster nebulosa* (Heteroptera: Pentatomidae), *L*. *equestris* (Heteroptera: Lygaeidae), and *C*. *marginatus* (Heteroptera: Coreidae) (Bayramova, 2015; Çetin, 2014; Demirkol, 2016; Kara, 2020; Metin, 2014). However, *P*. *apterus* and *Eurygaster* i*ntegriceps* (Heteroptera: Scutelleridae) midgut lacks gastric caecae (Al‐Sandouk, 1977; Mehrabadi et al., 2012).

They stated that there are abundant digestive aid bacteria in the gastric caeca in *C*. *pudicus* (Heteroptera: Pentatomidae), *D*. *baccarum* (Heteroptera: Pentatomidae), *R*. *nebulosa* (Heteroptera: Pentatomidae), *L*. *equestris* (Heteroptera: Lygaeidae), and *C*. *marginatus* (Heteroptera: Coreidae) (Bayramova, 2015; Çetin, 2014; Metin, 2014). However, no bacteria were observed in the gastric caeca in *E*. *spectabilis*.

### 4.5 Malpighian tubules and hindgut

Malpighian tubules vary greatly in number among species. There are four Malpighian tubules in *E*. *spectabilis*, as in *Triatoma infestans* (Heteroptera: Reduviidae) and *D*. *baccarum* (Mello & Dolder, 1977; Özyurt et al., 2017). While *E*. *integriceps* (Heteroptera: Scutelleridae) has 4–6 Malpighian tubules (Mehrabadi et al., 2012), *Solenopsis saevissima* (Hymenoptera: Formicidae), *C*. *mediterraneus* (Heteroptera: Pentatomidae) and stated that there are 6 Malpighian tubules in *C*. *tenebrionis* (Coleoptera: Buprestidae) (Arab & Caetano, 2002; Özyurt Koçakoğlu et al., 2020; Özyurt Koçakoğlu & Candan, 2022).

The color of the Malpighian tubules differs between species. In *E*. *spectabilis* the Malpighian tubules are white. However, in light yellow in *C*. *mediterraneus* (Özyurt Koçakoğlu & Candan, 2022). Also, in *P*. *apterus* (Heteroptera: Pyrrhocoridae), the proximal region of the Malpighian tubules appears brown, while the distal region appears green (Özyurt Koçakoğlu, 2021).

In histological examinations, the Malpighian tubules epithelial structure of *E*. *spectabilis* consists of a single‐layer cuboidal epithelial structure, as in *G*. *lineatum* (Amutkan et al., 2015). A similar structure was found in *P*. *apterus*, *C*. *mediterraneus*, and *A*. *rostrata* (Genç & Candan, 2024; Özyurt Koçakoğlu, 2021; Özyurt Koçakoğlu & Candan, 2022).

As in *P*. *apretus* and *C*. *mediterraneus* the hindgut of *E*. *spectabilis* consists of two parts, the pylorus and the rectum (Özyurt Koçakoğlu, 2021; Özyurt Koçakoğlu & Candan, 2022). However, in *Abedus ovatus* (Heteroptera: Belostomatidae), the hindgut includes ileum, rectal caeca, and rectum (Goverdhan et al., 1981).

Histologically, the shape of the cells surrounding the rectum. Different species have cylindrical, cubic, and flat shapes. The wall of the *E*. *spectabilis* rectum is surrounded by a single layer of cuboidal epithelium. The rectum of *P*. *apterus* by cuboidal epithelial cells (Özyurt Koçakoğlu, 2021). However, in *C*. *mediterraneus*, the rectal wall is single‐layered squamous epithelium (Özyurt Koçakoğlu & Candan, 2022).

Abundant uric acid crystals and bacteria were found in the rectum lumen of *E*. *spectabilis*. Similarly, they stated that uric acid crystals and many bacteria were seen in the lumen of *C*. *mediterraneus* (Özyurt Koçakoğlu & Candan, 2022). They also observed uric acid crystals in *C*. *pudicus*, *R*. *nebulosa*, *L*. *equestris*, *A*. *rostrata* (Bayramova, 2015; Demirkol, 2016; Genç, 2017; Metin, 2014).

In conclusion, our study shows that the alimentary canal of *E*. *spectabilis* presents different morphological and histological structures compared to those of other species. It reveals the similarities and differences regarding the alimentary canal. Thus, it aims to contribute to taxonomic and systematic studies. Additionally, this study provides additional information on which future researchers can build.

## AUTHOR CONTRIBUTIONS

**Hicret Arslan:** Conceptualization; data curation; investigation; validation; formal analysis; visualization; project administration; writing – review and editing; writing – original draft; resources. **Selami Candan:** Conceptualization; data curation; investigation; validation; formal analysis; visualization; project administration; writing – review and editing; writing – original draft; resources.

## Data Availability

The data that supports the findings of this study are available in the supplementary material of this article.
